# Academy of Natural Sciences

**Published:** 1842-01-22

**Authors:** 


### PROCEEDINGS OF SOCIETIES.

### Academy of Natural Sciences.

*Session of November* 16*th.* Vice President Morton in the Chair.

*Pigmy race of the Valley of the Mississippi.*

Dr. Morton made some remarks on the so called *Pigmy race* of people who are asserted to have formerly inhabited a part of the Valley of the Mississippi.

It has long been contended by intelligent persons, who, however, were ignorant of anatomy, that the adjusted bones of individuals of this race, never exceeded four feet and a half in height, and are often but three feet. These statements induced Dr. Morton to investigate the subject by means of a skeleton of one of these people, which he at length obtained through the kindness of Dr. Troost of Nashville; Mr. A. M'Call, a correspondent of Dr. Troost, having exhumed these remains from a cemetry near the Cumberland Mountain, in White county, Tennessee.

"The coffins," observes Mr. M'Call, in the letter read by Dr. Morton, "are from 18 to 24 inches in length, by 18 inches deep and 15 wide. They are made of six pieces of undressed sandstone or limestone, in which the bodies are placed with their shoulders and head elevated against the eastern end, and the knees raised towards the face, so as to put the corpse in a reclined or sitting posture. The right arm rested on an earthen pot, of about two pints in capacity, without legs, but with lateral projections for being lifted. With these pots, in some graves, are found basins and trays also of pipe clay and comminuted shells mixed; and no one of these repositories is without cooking utensils. In one of the graves was found a complete skull, and an os femoris, but most of the other bones were broken in hastily removing them. This is said to be the largest skeleton ever found at any of these burying grounds. It has the cranium very flat and broad, with very projecting front teeth, and appears to have pertained to an individual not over twelve or fourteen years of age."

After reading Mr. M'Call's letter, Dr. Morton exhibited the bones which accompanied it, and remarked that the stage of developement of the teeth, indicated a very juvenile subject. For example, many of the deciduous or first teeth yet remained in the jaws; while the only teeth of the permanent set which had protruded, were the first molars and the incisors, which, as every anatomist knows, make their appearance at about seven years of age. Of the other permanent teeth, some had no part formed but the crown, and all were completely embraced within the maxillary bones. The presence of the new incisors, isolated from the cuspidati which had not appeared, obviously gave rise to Mr. M'Call's remark respecting the very "projecting front reeth," but which, however, are perfectly natural in position and proportion. The cranial bones are thin, and readily separable at the sutures; nor does the *flat and broad* configuration of the cranium differ from what is common to the aboriginal American race. The long bones have their extremsties separated by ephiphyses; and every fact observed in these remains is strictly characteristic of early childhood; or about the seventh year of life. Even the recumbent or sitting posture in which they are found, has been observed in the dead bodies of the American nations from Cape Horn to Canada; and the utensils found with them, are the same in form and composition with those exhumed from the graves of the common Indians.

Dr. Morton concluded by remarking that these remains were to him an additional and convincing proof of what he had never doubted —viz. that the so called Pigmies of the western country, were merely children, who, for reasons not readily explained, but which actuate some religious communities of our own time, were buried apart from the adult people of their tribe.

